# Central circuit mechanisms of itch

**DOI:** 10.1038/s41467-020-16859-5

**Published:** 2020-06-16

**Authors:** Xiao-Jun Chen, Yan-Gang Sun

**Affiliations:** 1 Institute of Neuroscience, State Key Laboratory of Neuroscience, Center for Excellence in Brain Science & Intelligence Technology, Chinese Academy of Sciences, 320 Yue-Yang Road, 200031 Shanghai, China; 2 University of Chinese Academy of Sciences, 19A Yu-quan Road, 100049 Beijing, China; 3 Shanghai Center for Brain Science and Brain-Inspired Intelligence Technology, 201210 Shanghai, China

**Keywords:** Diseases of the nervous system, Somatosensory system

## Abstract

Itch, in particular chronic forms, has been widely recognized as an important clinical problem, but much less is known about the mechanisms of itch in comparison with other sensory modalities such as pain. Recently, considerable progress has been made in dissecting the circuit mechanisms of itch at both the spinal and supraspinal levels. Major components of the spinal neural circuit underlying both chemical and mechanical itch have now been identified, along with the circuits relaying ascending transmission and the descending modulation of itch. In this review, we summarize the progress in elucidating the neural circuit mechanism of itch at spinal and supraspinal levels.

## Abstract (web summary)

Itch is an important somatosensation, but the central mechanisms underlying it are not fully understood. Here, Chen and Sun review recent studies on the spinal and supraspinal circuits involved in itch processing.

## Introduction

Itch is defined as an unpleasant sensation that evokes a desire to scratch and consists of sensory, emotional, and motivational components^1^. Itch serves as an important protective mechanism that allows an animal to detect harmful substances invading the skin and remove them by scratching. The resultant scratching behavior, which is driven by strong emotional and motivational components, can sometimes induce a pleasant feeling, leading to an itch-scratch cycle. This itch-scratch cycle can result in serious skin damage for patients with chronic itch^2^.

Itch, like many other somatosensations, typically originates from the skin. The itch signals are relayed by peripheral sensory fibers to the spinal cord, where the information is processed by local interneurons before reaching the spinal projection neurons. The spinal projection neurons then send the itch signals to the brain via the ascending pathways, and further processing of itch sensation occurs in multiple brain areas and circuits^1,3–5^.

Based on peripheral inputs, itch can be classified into mechanical and chemical itch. Chemical itch can be further classified into histamine-dependent and histamine-independent subclasses according to the response to antihistamine agents^6^. Progress has been made in deciphering the molecular and cellular mechanisms of itch in the peripheral nervous system. Several key receptors, including members of the Mas-related G-protein-coupled receptor (Mrgpr) and serotonin receptor families, were found to be important for detecting chemical itch signals^7–12^. It was shown that MrgprA3 marks a group of itch-selective neurons in the dorsal root ganglia (DRG)^10^. In addition, transient receptor potential (TRP) channels have been shown to be recruited by histamine-dependent and histamine-independent itch pathways in the periphery for itch signal transduction^13,14^. Consistently, mutation of the TRP channels causes pathological itch^15^. More recently, it has been shown that sodium channels expressed in primary sensory neurons also play important roles in itch signal transmission^16,17^. These developments on the peripheral mechanisms of itch have been discussed in several excellent reviews^4,6,18,19^.

Using genetic, pharmacogenetic and optogenetic approaches, recent studies have also begun to dissect the itch circuitry within the central nervous system. In this review, we will focus on these central circuit mechanisms that contribute to the sensation of itch. Note that although it is not possible to determine if an experimental animal feels the sensation of itch, for the purposes of this review, we interchangeably use the terms itch and scratch for animal studies.

## Spinal circuits of chemical itch

**Fig. 1:**
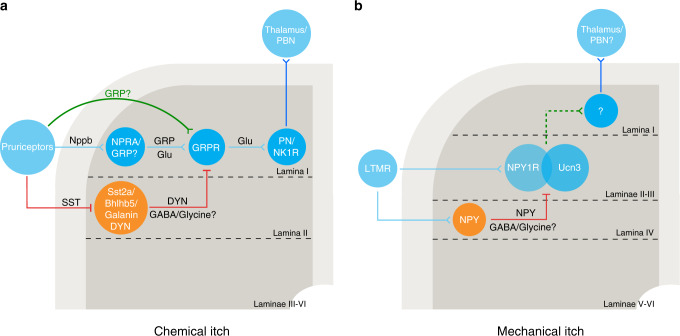
Model of spinal circuits for chemical and mechanical itch. **a** When activated by chemical itch stimuli, the peripheral pruriceptors send the itch signals to spinal NPRA^+^ neurons via Nppb, a primary itch transmitter. The secondary sensory (NPRA^+^ or GRP^+^) neurons then activate GRPR^+^ neurons by releasing GRP, after which the itch signals are conveyed to the spinal projection neurons before reaching the thalamus or PBN for further processing. GRP was also proposed as another peripheral itch transmitter in some studies. The chemical itch circuit is gated by spinal inhibitory interneurons marked by Bhlhb5, which also coexpress DYN and Sst2a. These neurons are inhibited by peripheral-derived SST, while in turn inhibiting itch via the release of DYN and GABA/glycine. Spinal galanin^+^ neurons also gate chemical itch processing by directly inhibiting the activity of GRPR^+^ neurons, and the majority of these inhibitory neurons overlap with DYN/Bhlhb5^+^ neurons. **b** Light touch stimuli activate LTMRs to evoke mechanical itch, and the mechanical itch information is transmitted to spinal Ucn3^+^ neurons, a subset of which also express NPY1R, and these neurons are gated by spinal inhibitory NPY^+^ neurons through NPY-NPY1R signaling as well as the inhibitory neurotransmitters GABA/glycine. PN projection neuron, LTMR low-threshold mechanoreceptor, Glu glutamate.

Multiple subtypes of excitatory neurons in the spinal cord are involved in itch processing. Genetic deletion of the transcription factor Tlx3 or testicular orphan nuclear receptor TR4 causes the loss of excitatory neurons in laminae I and II of the dorsal spinal cord, leading to significantly impaired pruritogen-induced scratching behavior^20,21^. Recent studies have identified the molecular markers for excitatory neurons involved in the processing of chemical itch. Spinal neurons expressing gastrin-releasing peptide receptor (GRPR) are predominantly excitatory neurons, representing a key component of the spinal itch circuit (Fig. 1a)^22,23^. This is evidenced by data showing that ablation of spinal GRPR^+^ neurons in mice almost completely abolishes the scratching behavior induced by both histamine-dependent and histamine-independent pruritogens^23^, while optogenetic or pharmacogenetic activation of GRPR^+^ neurons directly evokes itch-like scratching or biting behaviors^24^. Consistently, the number of spinal GRPR^+^ neurons are substantially decreased in mice lacking TR4, which show deficits in scratch responses^21^. In contrast, experimental ablation of spinal GRPR^+^ neurons does not affect acute pain behaviors, indicating that spinal GRPR^+^ neurons are selectively involved in processing itch signals. In addition, ablation of spinal GRPR^+^ neurons does not affect the number of neurons expressing neurokinin-1 (NK1R), which marks a significant percentage of projection neurons in the rodent spinal cord^23^. Consistently, studies have shown that there is limited overlap between spinal GRPR^+^ neurons and spinal projection neurons labeled by retrograde tracing from the ventral posterolateral or ventral posteromedial nucleus (VPL/VPM) of the thalamus or lateral parabrachial nucleus (LPBN)^25^. Moreover, it has been shown that spinal GRPR^+^ neurons form excitatory synapses with spinal projection neurons, which convey the itch signal to the brain for further processing^26^. Thus, GRPR^+^ neurons represent a subpopulation of excitatory interneurons in the dorsal spinal cord that selectively transmit itch information.

Mice lacking GRPR show impaired responses to different types of pruritogens^22^, suggesting that GRPR is necessary for itch at the spinal level. Using slice electrophysiology and optogenetics, Pagani et al. further explored the mechanism underlying GRP/GRPR neurotransmission^27^. They found that single action potentials in presynaptic GRP^+^ neurons were not sufficient to evoke action potentials in postsynaptic excitatory GRPR^+^ neurons, although these two neuronal types were monosynaptically connected via glutamatergic synapses at a very high connection rate. Only when the GRP^+^ neurons were activated in burst firing mode could suprathreshold activation of postsynaptic GRPR^+^ neurons be achieved, and this depolarized or active state of GRPR^+^ neurons persisted for minutes even after the presynaptic optogenetic stimulus was terminated. Furthermore, the progressive depolarization of GRPR^+^ neurons depends on GRPR but not glutamate receptor signaling, as blocking GRPR almost completely abolished the suprathreshold activation of GRPR^+^ neurons induced by burst-like stimulation of GRP^+^ neurons, while antagonizing the glutamate receptors did not.

Despite the essential role of GRP/GRPR in itch transmission, there has been long-standing controversy about the origin of GRP^28^. Early studies using immunohistochemistry showed that GRP was expressed in a subset of DRG neurons^22,29^, and the expression level of GRP was upregulated in DRGs in chronic itch models^30,31^. Dorsal root rhizotomy almost completely abolished GRP immuno-signals in the ipsilateral spinal cord^30^, confirming that the spinal GRP^+^ signals mainly originated from DRGs. However, some studies failed to detect GRP immunostaining signals in the DRG; instead, they found abundant GRP expression in the spinal cord. In addition, neither dorsal rhizotomy nor ablation of spinal cord TRPV1^+^ terminals altered GRP immuno-signals in the spinal cord^28^. Measuring GRP mRNA level by qPCR and ISH also confirmed that GRP was localized in the superficial dorsal spinal cord but not in the DRG^28,32^. Other groups further reported that GRP mRNA was barely detectable in DRGs of both juvenile and adult mice; instead, the majority of GRP is synthesized locally in the dorsal spinal cord^33^. Moreover, directly labeling the GRP^+^ neurons by using the GRP-EGFP or GRP-Cre transgenic mouse line confirmed the existence of GRP^+^ neurons in the dorsal spinal cord but not in the DRG^27,32,34–37^. Transcriptional profiling of DRG neurons using qRT-PCR or RNA-seq has revealed in an unbiased manner the molecular diversity of neurons underlying different somatosensation, yet GRP was not detected in these primary sensory neurons in several cases^38,39^, further supporting the idea of GRP expression in the spinal cord rather than in the DRG.

However, a recent study in which a GRP-Cre mouse line was generated using a knock-in strategy showed Cre^+^ signals in a subset of DRG neurons as well as in the spinal cord^40^. Conditional deletion of GRP in primary sensory neurons significantly attenuated itch behaviors, supporting the idea that GRP expression in primary sensory neurons is essential for itch signaling. This study also showed that there are GRP^+^ neurons in the dorsal spinal cord, the ablation of which does not affect itch or pain responses. Optogenetic activation of GRP^+^ fibers in the skin was found to evoke itch-like scratching behavior, further verifying the existence of GRP^+^ neurons in the DRG, as well as their functional role in itch transmission^40^. Taken together, these data strongly suggest that GRP exists in spinal excitatory interneurons, as well as peripheral sensory neurons, both of which act upon downstream spinal GRPR^+^ neurons for itch transmission. The differential observation of GRP expression in the DRG in different studies may have resulted from the different efficiency and specificity of GRP-Cre mouse lines, since different strategies were used to generate these mice. Nevertheless, the question of whether GRP is expressed in the DRG has yet to be fully resolved.

The functional role of spinal GRP^+^ neurons has been extensively studied. Ablation of spinal GRP^+^ neurons labeled in a GRP-Cre transgenic mouse line reduced pruritogen-induced scratching behavior, while pharmacogenetic activation of these neurons directly elicited spontaneous scratching behavior and enhanced the behavioral responses to various pruritogens^36^. By contrast, these manipulations caused no significant effect on the behavioral responses to acute noxious stimuli, emphasizing the selective role of spinal GRP^+^ neurons in itch. Consistent with the properties of the GRP/GRPR signaling pathway, in vivo optogenetic stimulation of spinal GRP^+^ neurons in burst mode, but not low-frequency stimulation with single pulses, elicited itch-like aversive behavior in freely moving mice^27^. Consistent with these findings, Sun et al. also showed that ablation of spinal GRP^+^ neurons decreased itch responses. However, they found that manipulation of spinal GRP^+^ neurons also affected pain responses^37^. Despite these conflicting results regarding the role of spinal GRP^+^ neurons in pain, which could largely result from the different experimental approaches for manipulating this neuronal population in these two studies, they both suggest that spinal GRP^+^ neurons play a critical role in spinal itch transmission (Fig. 1a). However, using a different mouse line, Barry et al. found that the ablation of spinal GRP^+^ neurons did not affect itch or pain responses^40^. This is likely due to differential labeling efficiency of the spinal cord neurons resulting from different mouse lines used in these studies. Thus, additional studies are needed to further address the functional role of spinal GRP^+^ neurons in itch.

In addition to GRP^+^ and GRPR^+^ neurons, another major component of the spinal circuit underlying chemical itch has also been identified^32^. Ablation of neuropeptide natriuretic polypeptide b (Nppb) receptor (Npra)-expressing neurons in the dorsal spinal cord also selectively eliminates the itch responses induced by pruritogens, suggesting an important role of Npra^+^ neurons in itch processing. This manipulation suppresses Nppb-evoked scratching behavior but does not affect scratching behavior evoked by intrathecal injection of GRP. Moreover, Npra is coexpressed with GRP in a subset of spinal interneurons^32^. These data suggest that Npra^+^ neurons are located upstream of GRPR^+^ neurons along the spinal itch pathway (Fig. 1a).

Another potential contributor to itch is somatostatin (SST), which is expressed in both the primary sensory neurons and the spinal cord. Mice lacking SST in both the peripheral and spinal cord exhibit behavioral deficits in response to pruritic stimuli. In addition, activation of the SST receptor Sst2a in the spinal cord enhances itch responses evoked by Nppb or GRP^41^. These data indicate that SST may also play an important role in modulating the spinal itch circuit.

## Gating the spinal circuits of chemical itch by local inhibitory neurons

Spinal inhibitory interneurons play a prominent role in regulating the spinal itch circuitry^42–44^. Dysregulation of spinal inhibitory interneurons could lead to hyperactivity of the itch circuit. This is seen from studies of mutant mice lacking the atonal-related transcription factor Bhlhb5, which is transiently expressed in the dorsal spinal cord^45^. These mice exhibit excessive scratching behavior and elevated itch responses to different types of pruritogens. Mice lacking Bhlhb5 were found to exhibit a dramatic loss of spinal interneurons expressing galanin and neuronal nitric oxide synthase (nNOS), but the distribution of two other inhibitory neuronal populations, expressing neuropeptide Y (NPY) or parvalbumin (PV), remained intact^46^, suggesting that spinal galanin^+^ and/or nNOS^+^ interneurons play a key role in gating chemical itch. Indeed, a more recent study examined the functional role of spinal galanin^+^ and nNOS^+^ neurons in itch signal processing and found that galanin^+^ neurons form predominant inhibitory synapses with spinal GRPR^+^ neurons and play an important role in gating chemical itch. By contrast, the functional role of spinal nNOS^+^ neurons in itch signal processing remains elusive, given the heterogeneous composition of this molecularly defined neuronal population, as well as their complicated synaptic connection with GRPR^+^ neurons^47^.

Furthermore, genetic deletion of vesicular glutamate transporter type 2 (*Vglut2*) in Nav1.8-expressing nociceptors resulted in pain deficits and enhancement of itch responses^48^, supporting the notion that pain can suppress itch at the spinal level^49^. Consistently, electrophysiological results in the monkeys have shown that scratching suppresses the neural activity of spinal neurons induced by pruritogens^50^, indicating an interaction between pain and itch at the spinal level. This finding is further supported by a recent study indicating that local inhibitory neurons might be involved in the interaction between itch sensation and other somatosensory stimuli in the spinal cord of the rodents^51^. The Bhlhb5^+^ interneurons likely receive direct synaptic input from primary sensory neurons responding to nociceptive and cooling stimuli^46^. Thus, Bhlhb5^+^ interneurons are well positioned to mediate the suppressing effect of pain on itch.

Spinal Bhlhb5^+^ neurons also express opioid peptide dynorphin (DYN)^46^. It was shown that kappa opioid receptor (KOR) agonists selectively inhibit itch-induced scratching behavior but not pain-related behaviors, while blocking KOR signaling by KOR antagonists increases pruritogen-induced itch responses. These results suggest that spinal Bhlhb5^+^ interneurons inhibit itch through kappa opioid signaling and that DYN acts as an important neuromodulator of itch transmission (Fig. 1a). Furthermore, intrathecal injection of a kappa opioid receptor agonist was found to attenuate GRP-induced scratching behavior, suggesting that DYN might modulate itch signal processing by acting on GRPR^+^ neurons directly, or downstream of GRPR^+^ neurons in the spinal cord^46^. Consistently, the application of a KOR antagonist evoked robust scratching behavior, and scratching was greatly reduced after ablating spinal GRPR^+^ neurons but not Npra^+^ neurons. These results indicate that DYN acts downstream of Npra^+^ neurons, probably at the level of spinal GRPR^+^ neurons^41^.

Moreover, pharmacogenetic activation of spinal DYN^+^ neurons significantly impairs pruritogen-induced scratching behavior, although such manipulation also affects mechanical nociception^41^. Thus, spinal DYN^+^ neurons also play a critical role in gating itch processing. Moreover, spinal DYN^+^ neurons extensively colocalize with galanin rather than nNOS^52^, and chemogenetic activation of nNOS^+^ neurons has no effect on pruritogen-induced behaviors, while modulating nociceptive responses. These data suggest that it is the DYN/galanin^+^ subpopulation of neurons, rather than the nNOS^+^ subpopulation of Bhlhb5^+^ neurons, that is likely to be more important for itch suppression, which is also consistent with recent findings that spinal galanin^+^ neurons gate chemical itch transmission^47^. However, another group reported that ablation of DYN^+^ neurons altered mechanical sensation but did not change pruritogen-induced scratching behavior^53^. This discrepancy is likely due to the difference in the strategies used to manipulate DYN^+^ neurons.

Spinal inhibitory neurons release gamma-aminobutyric acid (GABA) and/or glycine to tightly control the activity of postsynaptic neurons. Pharmacological activation of spinal α2 and α3 GABA_A_ receptors suppresses both acute and chronic itch, and chemogenetic activation of cervical spinal GABAergic neurons almost completely abolishes pruritogen-induced scratching behavior, indicating a powerful inhibitory control of spinal itch circuits by GABAergic neurons^47,54^. Similarly, pharmacogenetic activation of spinal glycinergic neurons was found to reduce neuropathic pain as well as pruritogen-evoked behaviors, indicating that spinal glycinergic neurons could also modulate the spinal itch circuitry^55^. Different molecularly identified subpopulations of spinal Bhlhb5^+^ neurons use GABA and/or glycine as fast neurotransmitters; thus, both GABAergic and glycinergic interneurons provide substantial inhibitory control of itch signal transmission in the spinal cord.

## Spinal circuits of mechanical itch

In addition to the chemical itch evoked by various pruritogens, itch sensation can also be evoked by light tactile stimulation, known as mechanical itch. In the periphery, mechanical stimuli are transduced and transmitted primarily by Merkel cells and Aβ primary sensory fibers, and the mechanosensitive Piezo channels expressed in Merkel cells functioning as the key mediator of mechanosensation^56,57^. Mechanical itch is independent of spinal GRPR^+^ neurons^58^, indicating that chemical itch and mechanical itch are processed by independent neural circuits at the spinal level.

Recent studies have identified several key components of the neural circuit for mechanical itch. It has been shown that spinal excitatory interneurons expressing urocortin 3 (Ucn3) represent a key node of the neural circuit for mechanical itch^59^. Ablation or pharmacogenetic inactivation of Ucn3^+^ neurons significantly attenuates the scratching behavior evoked by light touch stimuli but not by pruritogens. Consistent with the critical role in mechanical itch transmission based on behavioral experiments, Ucn3^+^ neurons receive synaptic input from peripheral myelinated Toll-like receptor 5 (TLR5^+^) low-threshold mechanoreceptors. Another subpopulation of spinal excitatory neurons, which express the neuropeptide Y1 receptor (NPY1R), was also shown to be critical for mechanical itch^60^. These neurons receive extensive input from cutaneous low-threshold mechanoreceptors. Ablation or pharmacogenetic silencing of spinal NPY1R^+^ neurons reduces mechanical itch-dependent scratching behavior but not scratching behavior evoked by pruritogens^60^. Consistently, activation of spinal NPY1R^+^ neurons increases light touch-induced as well as spontaneous scratching behaviors, which are both GRPR^+^ neuron-independent. Thus, NPY1R^+^ neurons are largely specialized for transmitting mechanical itch information. Interestingly, there is only partial overlap between NPY1R^+^ neurons and Ucn3^+^ neurons in the dorsal spinal cord^59^. However, another study found that intrathecal administration of a NPY1R agonist attenuated the scratching behavior evoked by both mechanical and histamine-dependent chemical itch^61^, indicating a partially overlapping pathway for the transmission of these two different itch submodalities. These results emphasize the complexity of the spinal circuits underlying mechanical itch and chemical itch.

The spinal mechanical itch circuit is also gated by local inhibitory neurons^58^. Ablation of spinal neurons labeled by neuropeptide Y (NPY) was found to induce spontaneous scratching behavior, which could not be blocked by the depletion of spinal GRPR^+^ neurons. Moreover, pharmacogenetic inactivation of spinal NPY^+^ neurons also enhanced light touch-induced scratching behavior^58^. These results support the idea that NPY^+^ inhibitory neurons in the spinal cord play a key role in gating the neural circuit responsible for mechanical itch. Consistently across studies, it has been shown that NPY^+^ neurons form functional inhibitory synaptic connections with NPY1R^+^ neurons and Ucn3^+^ neurons in the dorsal spinal cord^59,60^, confirming that NPY^+^ neurons gate the mechanical itch circuit at the spinal level (Fig. 1b).

## Transmission of itch signals from the spinal cord to the brain

Spinal projection neurons, which target multiple brain regions, serve as a key relay for sending various somatosensory information to the brain^1,62–65^. Among different pathways, both spinothalamic and spinoparabrachial pathways are involved in the transmission of itch signals (Fig. 1). The transmission of itch signals from the spinal cord to the thalamus has been shown by electrophysiological studies in different animal species showing that spinothalamic and trigeminothalamic tract neurons are activated by peripheral pruritic stimuli^66–68^, which is consistent with the data showing that the number of c-Fos-positive spinal projection neurons increases after application of pruritogens to the skin^26^. Interestingly, different types of pruritogens activate distinct subsets of spinothalamic tract (STT) neurons in primates^67^, suggesting that histamine-dependent and histamine-independent itch are processed by distinct pathways.

The functional role of spinal projection neurons has also been explored with behavioral experiments in rodents. The majority of projection neurons in the superficial dorsal horn express the neurokinin 1 receptor (NK1R)^62,69,70^, which is the receptor for substance P. Ablation of spinal NK1R^+^ neurons by intrathecal application of substance P-saporin significantly reduces scratching behavior evoked by pruritogens as well as chronic itch^71,72^. Interestingly, ablation of spinal NK1R^+^ neurons does not affect mechanical itch responses^60^, supporting the segregation between chemical and mechanical itch pathways. Elimination of spinal NK1R^+^ neurons also attenuates behavioral responses to noxious stimuli^72,73^, suggesting that nociceptive signals are also conveyed by these projection neurons. Electrophysiological recording revealed that primate STT neurons respond to peripheral itch, pain, mechanical, and thermal stimuli^67^, indicating the polymodal properties of spinal projection neurons. Information from different somatosensory modalities is thus processed and integrated by spinal projection neurons^1,43^.

Electrophysiological recording experiments in the rats also demonstrated that the majority of trigeminoparabrachial tract (VcPbT) neurons could be activated by various pruritogens^74^, similar to the response properties of trigeminothalamic tract (VcTT) neurons. Unlike VcTT neurons, the VcPbT neurons showed a delayed peak as well as a prolonged response pattern after application of 5-hydroxytryptamine (5-HT)^74^, which better matched the time course of behavioral responses to pruritogens in awake animals. It was recently discovered that the spinoparabrachial pathway is activated by peripheral pruritic stimuli and that optogenetic inhibition of this pathway attenuated pruritogen-induced scratching behavior^26^, suggesting that the spinoparabrachial pathway plays a critical role in processing itch information. The parabrachial nucleus (PBN)-projecting spinal projection neurons receive direct synaptic inputs from spinal GRPR^+^ neurons^26^, suggesting that itch signals are transmitted from GRPR^+^ neurons to the PBN via a disynaptic circuit. Thus, PBN serves as a first central relay for itch transmission in the brain.

As mentioned earlier, ascending transmission of distinct somatosensory modalities, i.e., pain and itch, relies on spinal projection neurons. The interaction between pain and itch is widely distributed along sensory pathways, and there has been limited evidence directly examining the functional roles of spinocerebral projections in pain and itch simultaneously. It is thus possible that pain and itch recruit a similar population of spinal projection neurons. Whether these projection neurons can be further genetically and functionally divided into subclasses remains to be explored.

## Brain areas involved in itch processing

The representation of itch signal processing in the brain in earlier studies was mostly investigated by macroscopic imaging approaches, including positron emission tomography (PET) scans and functional MRI (fMRI). These functional imaging studies in humans have identified many brain regions that are activated by pruritic stimuli, as well as by itch-associated scratching or emotional changes^75–92^. Despite some differences in the brain regions revealed by different studies, most studies have found activation of the thalamus, primary and secondary somatosensory cortex (S1 and S2), prefrontal cortex (PFC), anterior cingulate cortex (ACC), insular cortex, premotor and motor cortex, and parietal cortex. These distinct brain areas are thought to be involved in different aspects of itch signal processing^1–3,92^. The thalamus is typically recognized as the relay station for somatosensation from the spinal cord to the primary sensory cortices, and the somatosensory cortex is proposed to encode the spatial, temporal, and intensity aspects of itch sensation^1–3,92^. Motor areas are thought to be involved in the planning and execution of itch-induced scratching behavior. Higher-order cortices, including the PFC and ACC, are likely involved in processing the emotional or motivational components of itch sensation. In rodents, by anterograde tracing initiated from itch-selective spinal GRP^+^ neurons^36^, several brain areas, including the thalamus, PBN, amygdala, S1, periaqueductal gray (PAG), and rostral ventromedial medulla (RVM), have been suggested to be itch-related brain areas. However, the functional role of most of these brain areas in itch remains to be examined.

Histamine-dependent and histamine-independent itch activate slightly different brain regions, although a series of core brain areas are activated by both kinds of itch, as revealed by human functional imaging studies^87^. Histamine-independent itch induced by cowhage more extensively recruits the insular cortex, claustrum, basal ganglia, thalamic nuclei and pulvinar. Histamine-dependent and histamine-independent itch are thus processed by largely overlapping yet distinct neural networks. The brain activity patterns responding to itch and pain are very similar^88^. No itch-selective brain region has been identified in most studies, suggesting that the differences between itch and pain signal processing possibly exist at the cellular level.

Molecular and cellular research strongly supports the existence of labeled lines, at least at the level of peripheral sensory fibers and spinal cord interneurons, by which different somatosensory modalities are processed by specific receptors, cells or neuronal circuits^4,5^. Electrophysiological studies, however, have directly challenged this theory by showing multimodal response properties of neurons at different stages along the sensory pathways to different types of stimuli^66,93^. Instead, these data point toward a population coding mechanism that underlies the generation of the specific quality of somatosensation^49,94,95^. It is challenging to reconcile the data obtained in behavioral and electrophysiological experiments, since the experimental conditions, animal species, investigative strategies and approaches were all discrepant in different studies, yet it is hard to resist the idea that parallel pathways do exist to process specific somatosensory information, especially at the level of DRG and spinal interneurons. Given the complex interactions between itch and pain, or between other somatosensations at multiple neural levels, plus the fact that similar brain structures and neural networks could be recruited by both itch and pain, population coding might be a more economical and efficient way to differentiate diverse sensory modalities in the central nervous system, especially at the level of spinal projection neurons and higher brain centers, although we cannot completely exclude the possibility that dedicated labeled lines are retained at the supraspinal level.

**Fig. 2:**
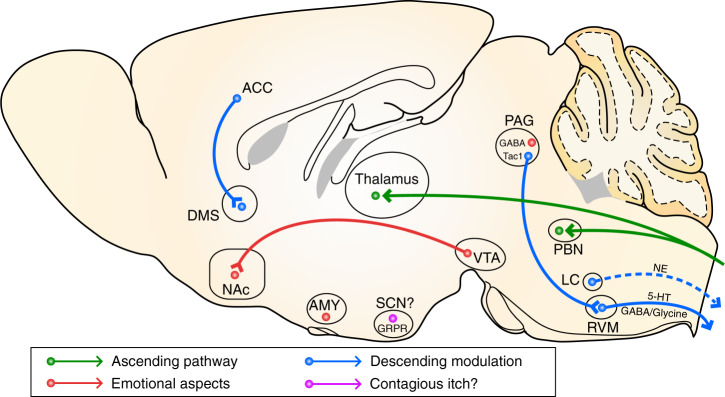
Brain circuits for itch signal processing and modulation. The chemical itch signals are first relayed to the PBN and thalamus by spinal projection neurons, while visually contagious itch in mice is proposed to be mediated by the SCN GRPR^+^ neurons in the hypothalamus. The emotional components of itch sensation may be encoded by the amygdala, GABAergic neurons in the PAG, and different neuronal populations in the VTA. The PAG^Tac1^-RVM circuit is involved in the descending modulation of itch signal processing, and brain-derived neuromodulators such as 5-HT and noradrenaline can also substantially modulate spinal itch circuits in a feedback manner. Projections from the ACC to the DMS selectively modulate histamine-dependent itch. AMY amygdala, LC locus coeruleus, NE noradrenaline.

During the past few years, tremendous progress in the development of optogenetics, electrophysiology, in vivo imaging and other techniques has led to an unprecedented opportunity to functionally dissect the role of different brain areas in itch signal processing. It has been shown that PBN neurons are activated by pruritic stimuli and that pharmacogenetic inactivation of PBN neurons significantly impairs itch-induced scratching behavior^26^. Furthermore, selective knockout of the glutamate transporter *Vglut2* in the PBN significantly decreases the scratching behavior evoked by various types of pruritogens, as well as the scratching behavior in an allergic itch model, which suggests that PBN glutamatergic neurons play an important role in itch processing and further verifies the essential role of PBN in processing the itch signal (Fig. 2). PBN sends dense projections to the amygdala, and it was shown that acute inactivation of the central amygdala (CeA) by infusion of a GABA_A_ agonist reduced pruritogen-induced scratching behavior^96^, suggesting that CeA is also involved in itch processing.

Dopaminergic (DA) neurons in the ventral tegmental area (VTA) play a key role in encoding motivational drive^97^. It was shown that VTA DA neurons are important for driving itch-induced scratching behavior^98^. The activity of the DA neuronal population was immediately elevated after the onset of itch-induced scratching behavior, as recorded with fiber photometry in mice. This activation pattern of VTA DA neurons was also verified by single-unit electrophysiological recording of opto-tagged DA neurons, in which a significant proportion of VTA DA neurons increased their firing rate even slightly before the scratching onset, suggesting that VTA DA might encode the driving force for scratching behavior. Indeed, optogenetic inactivation of VTA DA neurons impaired pruritogen-induced scratching behavior. This is consistent with previous pharmacological studies, in which blocking dopamine D1 receptors suppressed the scratching behavior induced by compound 48/80^99^. Moreover, activation of dopamine D2, but not D1 receptors, directly induced scratching behavior in monkeys^100^, further indicating that DA signaling could promote scratching behavior. Yuan et al. also investigated the activity of DA projections from VTA to the nucleus accumbens (NAc) during itch-induced scratching behavior^98^ and found that DA fibers in the lateral shell of the NAc (NAc LaSh) showed more enhanced activity after scratching onset than in the medial shell of the NAc, suggesting that the VTA-NAc LaSh projection might be more prominently involved in itch-induced scratching behavior.

## Brain mechanisms of contagious itch

Watching others scratching themselves or even talking about itchiness can induce a desire to scratch in humans, despite no chemical or mechanical pruritic input^1,91^. This phenomenon is referred to as contagious itch. Previous fMRI studies have found that visually induced itch activates many brain regions that are typically involved in the processing of physical itch^91^, including the primary somatosensory cortex, insular cortex, and prefrontal cortex.

A recent study demonstrated that contagious itch behavior is also observed in mice^101^. Mice exhibiting contagious itch behavior show higher level of c-Fos expression, as well as lower level of GRP expression in the suprachiasmatic nucleus (SCN) of the hypothalamus, indicating that GRP^+^ neurons in the SCN may be involved in visually contagious itch^101^. The receptor of GRP is also highly expressed in the SCN, and conditional knockout of *Grpr* or ablation of GRPR^+^ neurons in the SCN reduces imitative scratching behavior. These data suggest a potential role of GRP–GRPR signaling in the SCN for processing visually contagious itch in mice (Fig. 2). However, other groups^102,103^ failed to reproduce the imitative scratching behavior in normal mice observing demonstrator mice receiving histamine application, nor did they detect significant temporally contiguous scratching in observer mice. In addition, the total scratching bouts during the entire observation period was also not different between observers and control mice^103^. Similarly, another study also failed to replicate contagious scratching in mice using an itching video-based paradigm, whereas itch sensation and responses were successfully elicited in humans^102^. Thus, whether mice can serve as a good animal model for studying contagious itch sensation is still debatable. Nevertheless, the central circuit mechanisms of contagious itch are much less explored, and it would be interesting and important to investigate the neural basis of contagious itch, especially in more sophisticated animal models such as primates.

## Emotional components of itch sensation

Itch, like many other somatosensations, is a complex experience consisting of sensory, emotional and motivational components. Indeed, animal studies have shown that acute pruritic stimuli can induce conditional place aversion (CPA), as well as anxiety-like behaviors, as revealed by the open field test (OFT) and elevated plus maze (EPM) assay, suggesting a negative emotional component of itch sensation^104–106^. As indicated by previous human functional imaging studies, multiple brain regions could be involved in processing the affective components of itch sensation^1,2^.

Recent animal studies have further explored the possible mechanism underlying the negative emotional component of itch sensation. A subpopulation of CeA neurons was activated by various types of pruritogens, and a subset of these itch-responsive neurons also responded to noxious stimuli^104^. Moreover, the neuronal activity of amygdala-projecting brain areas such as the PBN and mPFC was also increased after pruritogen injection^104^. Importantly, optogenetic activation of histamine-responsive amygdala neurons reduced the duration in the open arm of the EPM and center time in the OFT, suggesting that the amygdala plays a critical role in encoding the negative emotional component of itch sensation. In addition, VTA GABAergic neurons also play a critical role in encoding the aversive component of itch sensation. The activity of VTA GABAergic neurons is immediately increased after itch-induced scratching behavior^105^. Silencing VTA GABAergic neurons has been found to impair itch-associated CPA^105^, supporting the prominent role of VTA GABAergic neurons in encoding the negative emotional aspect of itch sensation. Additionally, a recent study demonstrated that PAG is critical for modulating the affective component of itch. Pharmacogenetic activation of PAG GABAergic neurons impairs itch-associated CPA, suggesting that PAG GABAergic neurons modulate the affective component of itch^107^. At the spinal level, the negative emotional signal associated with itch is conveyed by GRPR^+^ neurons^106^.

Although itch is an unpleasant sensation, scratching an itch can produce a hedonic experience^50^, which could facilitate the scratching behavior. VTA is a widely recognized reward center in the brain and is capable of encoding both value and salience^97^. Human functional imaging studies have indicated that VTA is involved in the pleasure associated with scratching an itch^84,90^, and this was further tested in a recent animal study^105^. Recording the population activity of DA neurons in the mouse VTA in response to pruritogen-induced scratching behavior with fiber photometry showed that acute pruritic stimuli activated VTA DA neurons with a several-second delay upon scratching onset. Moreover, the activation of DA neurons vanished during scratching attempts when the scratching behavior after pruritogen application was prevented with a custom-made collar, suggesting that VTA DA neurons might signal the pleasure associated with scratching an itch^105^. Furthermore, inactivating VTA DA neurons attenuated scratch-associated conditional place preference (CPP), further supporting the crucial role of the VTA in regulating the emotional components of the itch-scratch cycle (Fig. 2). The response of DA neurons exhibited high heterogeneity^108^, which could contribute to their functional heterogeneity^98,105^. Further molecular or circuit-based dissection of VTA DA neurons in itch sensation is required.

## Neural circuits underlying modulation of itch signal processing

The processing of itch is gated by top-down modulation, and multiple brain areas can differentially modulate itch signal processing. Human functional imaging studies have shown that the activity of PAG is altered during itch processing^2,87,89^. Recordings from the PAG using fiber photometry in mice have also shown that the activity of PAG increases during itch processing^107,109^. Given its critical role in the descending modulation of pain, it has been proposed that PAG could also play an important role in modulating itch^1,49,92^. This is supported by an animal study showing that electrical stimulation of the PAG suppresses histamine-evoked responses in spinal dorsal horn neurons^93^.

Recent studies examined the functional role of multiple neuronal subtypes of PAG in itch and found that glutamatergic and GABAergic neurons in the PAG are differentially involved in itch as well as pain regulation^107,109,110^. Moreover, a subpopulation of PAG glutamatergic neurons expressing tachykinin 1 (Tac1) plays an important role in facilitating the itch-scratch cycle, since the ablation or pharmacogenetic inhibition of these neurons reduces itch-induced scratching behavior. Pharmacogenetic or optogenetic activation of PAG Tac1^+^ neurons has been found to directly induce a robust itch-like scratching behavior. The scratching behavior induced by activation of Tac1^+^ neurons is reduced by ablation of spinal GRPR^+^ neurons, suggesting that PAG Tac1^+^ neurons modulate the spinal itch processing through a descending pathway^109^. PAG Tac1^+^ neurons form glutamatergic synapses with spinal cord-projecting RVM neurons, supporting the idea that the PAG^Tac1^-RVM circuit plays an important role in the descending regulation of itch sensation (Fig. 2).

The neuromodulatory system plays important roles in the descending modulation of spinal itch signal processing^5^. Among all neuromodulators, 5-HT has been most widely implicated in the descending control of spinal sensory processing. Serotoninergic neurons in the RVM send projections to the trigeminal nucleus caudalis and the dorsal horn of the spinal cord. Depletion of 5-HT^+^ fibers in the spinal cord decreases pruritogen-induced scratching behavior^111^, suggesting that descending serotoninergic projection facilitates itch processing in the spinal cord. This effect is largely mediated by 5-HT1A receptors, which are coexpressed with GRPR in some spinal cord neurons. Coactivation of 5-HT1A and GRPR increases the excitability of spinal GRPR^+^ neurons and greatly potentiates GRP-GRPR signaling.

Interestingly, the facilitation of the spinal itch circuit by PAG Tac1^+^ neurons is not mediated by descending serotoninergic projections^109^, indicating the existence of parallel pathways originating from RVM for itch modulation. Indeed, RVM can also form GABA/glycine-mediated fast inhibitory synapses with spinal GRPR^+^ neurons, providing another potential descending pathway for itch modulation^47^. However, this finding cannot explain the facilitation of itch by the PAG^Tac1^-RVM circuit, as those projections are excitatory^109^. One possibility is that RVM modulates the spinal itch circuit through multiple pathways, and RVM neurons could facilitate itch signal processing by a disynaptic disinhibition mechanism via local spinal inhibitory neurons. The essential role of the RVM in the descending modulation of pain has also been extensively studied^112,113^. However, the exact identity of RVM neurons responsible for pain or itch modulation is still unknown, and it remains to be elucidated whether the descending modulation of itch or pain is achieved by distinct subpopulations of RVM neurons or whether it is a shared neuron ensemble exerting a more generalized modulation in the processing of various types of somatosensory information. In addition, noradrenaline is also involved in the regulation of spinal itch signal processing^114–116^. However, the circuit mechanism underlying the modulation of the spinal itch circuitry by locus coeruleus-derived noradrenaline remains to be further examined.

Higher-order brain areas can also dynamically modulate itch processing. It has been shown that the projections from ACC to the dorsomedial striatum (DMS) selectively modulate histamine-dependent itch processing^117^. DMS-projecting ACC neurons are activated by histamine; and disruption of the ACC–DMS pathway by targeted regional lesions attenuates histamine-induced but not 5-HT-induced scratching behavior, and does not affect the responses to acute noxious stimuli. In line with these results, synaptic transmission in the ACC was shown to be potentiated by chronic itch^118^. Furthermore, optogenetic inhibition of the ACC–DMS connections suppressed the scratching behavior induced by histamine-dependent but not histamine-independent pruritic stimuli and did not change capsaicin-evoked nocifensive behaviors. Moreover, optogenetic activation of this pathway induced spontaneous scratching, which was greatly abolished after antagonizing histamine H1 or histamine H4 receptors^117^. These data indicate that the ACC–DMS circuit exhibits a selective modulatory role in histaminergic itch (Fig. 2), further demonstrating the existence of segregated cerebral circuits mediating histamine-dependent and histamine-independent itch sensation.

## Future directions

During the past decade, tremendous progress has been made in deciphering the circuit mechanisms of itch sensation in both the spinal cord and the brain^5^. Several dedicated neural circuits have been demonstrated to process different forms of itch as well as modulate different aspects of itch sensation. Despite all these exciting advances in the itch field, several key questions remain to be addressed.

There is still debate about the coding mechanism of itch^1,42,43,94,95^. How is itch discriminated from other somatosensations? Where is the perceptual distinction between different somatosensory modalities achieved? To address these questions, it will be important to record the activity of neurons with cellular resolution along the sensory pathways during itch processing in awake animals, which can be achieved by in vivo extracellular recording or calcium imaging with endoscopy or miniaturized two-photon microscopy^119,120^.

Multiple key components of the neural circuit for itch signal processing have been identified at the spinal level. However, understanding of the spinal circuit for itch processing and modulation is not complete. We know more about the inhibitory neuronal control of transmission for chemical itch, but less is known about the roles of different inhibitory neuronal populations in the regulation of mechanical itch^5,59,60^. Whether those circuits actively involved in chemical itch modulation can also provide substantial modulation of mechanical itch or whether these two different forms of itch sensation are dynamically regulated by distinct inhibitory neuronal networks remains to be elucidated. How do different somatosensory modalities interact at the spinal level? The identity of the spinal projection neurons that send different types of itch information to the brain is still unknown. What is the functional difference between the spinothalamic and spinoparabrachial projections in conveying itch as well as other somatosensory information? Recent sequencing studies have provided more insight into the classification of neurons in the spinal cord^121,122^, and such knowledge together with new tools for dissecting local circuits will guide further dissection of spinal circuits. Furthermore, the PBN is demonstrated to be a first relay station for itch transmission in the brain^26^, yet the genetic identity of itch-responsive PBN neurons needs to be determined. Since PBN has been shown to participate in many other physiological processes, such as pain sensation^123^, it will be important to further investigate the integration or segregation of different sensory information in the PBN.

Finally, our knowledge of the modulatory network of itch sensation, especially the emotional and motivational components of itch, is still limited and requires further systematic investigation. It is also critical to gain a deep understanding of the circuit mechanism underlying the positive emotional component, which is one of the major driving forces for the vicious itch-scratch cycle. Developing new paradigms will be the key to address these questions. Additionally, the circuit mechanisms underlying chronic itch are largely unknown. Dissection of the mechanism of chronic itch will guide the development of new therapeutic approaches for itch.
